# Integrating mitoepigenetics into research in mood disorders: a state-of-the-art review

**DOI:** 10.3389/fphys.2024.1338544

**Published:** 2024-02-08

**Authors:** Deniz Ceylan, Hidayet Ece Arat-Çelik, Izel Cemre Aksahin

**Affiliations:** ^1^ Department of Psychiatry, School of Medicine, Koç University, Istanbul, Türkiye; ^2^ Koç University Research Center for Translational Medicine (KUTTAM), Affective Laboratory, Istanbul, Türkiye; ^3^ Department of Psychiatry, School of Medicine, Maltepe University, Istanbul, Türkiye; ^4^ Graduate School of Health Sciences, Koç University, Istanbul, Türkiye

**Keywords:** mood disorders, bipolar disorder, major depression, epigenetics, mitochondrial dysfunction, mitoepigenetics

## Abstract

Mood disorders, including major depressive disorder and bipolar disorder, are highly prevalent and stand among the leading causes of disability. Despite the largely elusive nature of the molecular mechanisms underpinning these disorders, two pivotal contributors—mitochondrial dysfunctions and epigenetic alterations—have emerged as significant players in their pathogenesis. This state-of-the-art review aims to present existing data on epigenetic alterations in the mitochondrial genome in mood disorders, laying the groundwork for future research into their pathogenesis. Associations between abnormalities in mitochondrial function and mood disorders have been observed, with evidence pointing to notable changes in mitochondrial DNA (mtDNA). These changes encompass variations in copy number and oxidative damage. However, information on additional epigenetic alterations in the mitochondrial genome remains limited. Recent studies have delved into alterations in mtDNA and regulations in the mitochondrial genome, giving rise to the burgeoning field of mitochondrial epigenetics. Mitochondrial epigenetics encompasses three main categories of modifications: mtDNA methylation/hydroxymethylation, modifications of mitochondrial nucleoids, and mitochondrial RNA alterations. The epigenetic modulation of mitochondrial nucleoids, lacking histones, may impact mtDNA function. Additionally, mitochondrial RNAs, including non-coding RNAs, present a complex landscape influencing interactions between the mitochondria and the nucleus. The exploration of mitochondrial epigenetics offers valuable perspectives on how these alterations impact neurodegenerative diseases, presenting an intriguing avenue for research on mood disorders. Investigations into post-translational modifications and the role of mitochondrial non-coding RNAs hold promise to unravel the dynamics of mitoepigenetics in mood disorders, providing crucial insights for future therapeutic interventions.

## Introduction

Mood disorders, such as bipolar disorder (BD) and major depressive disorder (MDD), are highly prevalent and profoundly disabling conditions. BD is specifically characterized by severe lows (depression) and elevated highs (hypomania or mania), whereas MDD includes only the depressive pole. Common symptoms often observed in depression, such as psychomotor retardation, reduced engagement in daily activities, difficulty in concentrating, decreased attention span, and slower processing speed, as well as symptoms in mania, such as increased psychomotor activity, increased talkativeness, rapid speech, reduced need for sleep, racing thoughts, and distractibility, suggest that disruptions in energy-related processes through mitochondrial abnormalities may underlie mood episodes (Stork and Renshaw, 2005; Karabatsiakis et al., 2014). This connection is supported by evidence indicating a high prevalence of MDD (37%–54%) and BD (17%) in individuals with mitochondrial diseases (Kasahara and Kato, 2018).

Mitochondria, recognized as the “powerhouses of cells”, have been linked to mood disorders. Neuronal functioning requires a high amount of energy; therefore, the brain utilizes 25% of the body’s energy substrates and consumes 20% of its oxygen (Wong-Riley et al., 1989; Zhu et al., 2012). As energy derived from anaerobic glucose is insufficient to sustain the brain’s energy metabolism without high-energy sources such as glycogen, proper mitochondrial metabolism becomes crucial, generating 92% of the body’s energy through oxidative phosphorylation. Mitochondria also play direct roles in processes crucial for neural functioning, such as calcium homeostasis, apoptosis, signal conduction, and neurogenesis, all of which have significant implications in mood disorders (Rizzuto et al., 2012; Bock and Tait, 2020). Additionally, various mechanisms that actively interact with mitochondrial processes, such as oxidative stress, inflammation, neuroplasticity, neurogenesis, and stress-related processes, are associated with the pathogenesis of mood disorders (Manoli et al., 2007; Miller, 2011; Bansal and Kuhad, 2016; Midzak and Papadopoulos, 2016; Culmsee et al., 2018; Konttinen et al., 2019; Lapp et al., 2019). Additionally, studies have indicated the impact of various psychotropic agents such as antidepressants and mood stabilizers on mitochondrial function (Lundberg et al., 2020). Proteomic studies have highlighted mitochondrial pathways (Föcking et al., 2016), and animal models of depression have revealed a connection between mitochondrial dysfunction and depression-like behaviors (Kolar et al., 2021). This highlights the current evidence, emphasizing the pivotal role of mitochondria as a vital research area in mood disorders (Manji et al., 2012; Morava and Kozicz, 2013; Allen et al., 2018; Kuffner et al., 2020).

The heritability of MDD has been estimated to be approximately 40%, and for BD is estimated to be approximately 70%, with first-degree relatives of individuals with mood disorders facing an elevated risk of mood disorders 2–3 times higher for depression and up to 10 times higher for BD (Sullivan et al., 2000; Penner-Goeke and Binder, 2019). Despite its high heritability, a specific gene that strongly contributes to mood disorders remains unidentified, and genome-wide association studies explain only a fraction of the genetic diversity in these disorders (Menke et al., 2012; Malhi and Mann, 2018). One of the major challenges to comprehending the progression of mood disorders is the lack of a single genetic, biological, or psychosocial explanation. Instead, these disorders are thought to result from the complex interplay of multiple factors. Individuals with mood disorders manifest symptoms that arise from a complex interplay of genetic and environmental factors, including childhood trauma, migration, loneliness, poverty, work stress, violence, and even air pollution (Zeng et al., 2019). The interplay between genetic and environmental factors in mood disorders is shown by epigenetic alterations (Penner-Goeke and Binder, 2019; Legrand et al., 2021), which play a role in mood disorders and influence the effects of antidepressants and mood-stabilizing agents (Menke et al., 2012; Mikhed et al., 2015; Saavedra et al., 2016).

Mitochondria are semi-autonomous organelles that contain their own, circular, maternally inherited genomes. While traditional epigenetic studies have primarily concentrated on the nuclear genome, recent research underscores that mtDNA undergoes epigenetic changes, leading to the emergence of new fields of mitochondrial epigenetics and mitoepigenetics (Ghosh et al., 2015; Cavalcante et al., 2020). Given the crucial role of mitochondrial dysfunction and epigenetic alterations in the pathogenesis of mood disorders, changes in the epigenetics of the mitochondrial genome have gained significance. In this review, we aim to compile and present existing data regarding epigenetic alterations in the mitochondrial genome in mood disorders, thereby establishing a foundation for future research on the origins of these conditions.

## Mitochondrial genome alterations and mood disorders

Mitochondrial DNA (mtDNA) encodes 13 polypeptides, 22 transfer RNAs (tRNAs), 2 ribosomal RNAs (rRNAs), and a non-coding region known as the displacement loop (D-loop). Unlike nuclear DNA, mtDNA is in the matrix of the mitochondria rather than the cell nucleus. It lacks histones but is organized into clusters coated with nuclear counterpart proteins, called nucleoids. Research in mtDNA modifications remains limited and mainly focuses on mtDNA alterations, reporting changes in both mtDNA copy numbers and oxidation levels (Wang et al., 2018; Bodenstein et al., 2019; Czarny et al., 2020b; Chung et al., 2022). On the other hand, mitochondrial epigenetics explores changes in mtDNA and the mitochondrial genome (Manev, 2014), whereas mitoepigenetics encompasses a broader scope, incorporating interactions between mtDNA and nuclear DNA.

MtDNA copy number is a measure of the quantity of mitochondrial genomes per cell and serves as a surrogate indicator of mitochondrial health. Numerous studies have investigated mtDNA copy numbers in various types of specimens, including brain slices, saliva, and blood, among individuals with mood disorders (Kakiuchi et al., 2005; Kim et al., 2011; Cai et al., 2015; Chang et al., 2015; Nicod et al., 2016; Lindqvist et al., 2018; Chung et al., 2019; Tsujii et al., 2019). In human *postmortem* studies, mtDNA copy number was found to be increased in the dorsolateral prefrontal cortex and decreased in the superior temporal gyrus in individuals with BD (Das et al., 2022). Wang et al. suggested that leukocyte mtDNA copy number was significantly lower in individuals with BD (even in mania or depression) than in healthy controls (Wang et al., 2018). Chung et al. found that mtDNA copy number decreased in individuals with BD type I but increased in individuals with BD type II compared to controls (Chung et al., 2022). On the other hand, Fries et al. showed that individuals with BD had elevated levels of mtDNA copy numbers compared to controls, while there was no significant difference between siblings of individuals with BD and controls (Fries et al., 2017). Despite some conflicting results, most of the literature suggests significant alterations in mtDNA copy numbers in MDD and BD (Czarny et al., 2020a).

During oxidative phosphorylation in mitochondria, approximately 5% of the oxygen consumed in the electron transport chain is transformed into reactive oxygen species (ROS), such as superoxide (O_2_^−^) and hydrogen peroxide (H_2_O_2_)). ROS can damage various components of the cell, including the mitochondrial and nuclear genomes. Owing to its proximity to oxidative phosphorylation and the absence of histone protection, mtDNA has been suggested to be three times more vulnerable to the effects of ROS than nuclear DNA (Yakes and Van Houten, 1997). To date, a very limited number of studies have focused on the oxidation of mtDNA, although increased oxidation of nuclear DNA has repeatedly been shown in mood disorders (Ceylan et al., 2018; Ahmadimanesh et al., 2019; Ceylan et al., 2020a; Czarny et al., 2020a; Kucuker et al., 2022; Çelik et al., 2023). Two clinical studies have reported increased mtDNA damage in peripheral samples of patients with unipolar depression (Chang et al., 2015; Czarny et al., 2020b). Chang et al. reported that individuals with MDD, even in remission, exhibited higher mitochondrial oxidative damage than healthy controls (Chang et al., 2015). Furthermore, Czarny et al. reported that individuals with depression showed elevated levels of mtDNA damage in peripheral blood mononuclear cells (PBMCs) compared to controls, whereas there was no alteration in mtDNA copy number (Czarny et al., 2020b). On the other hand, a post-mortem study showed a significant decrease in oxidative mtDNA damage in the brain slices of patients with BD (Bodenstein et al., 2019). In addition, a recent study showed that oxidative mtDNA damage may induce short- and long-term immune activation (Xian and Karin, 2023). While these findings may not be conclusive on their own, it can be posited that oxidatively induced mtDNA damage plays an important role in mood disorders.

**FIGURE 1:**
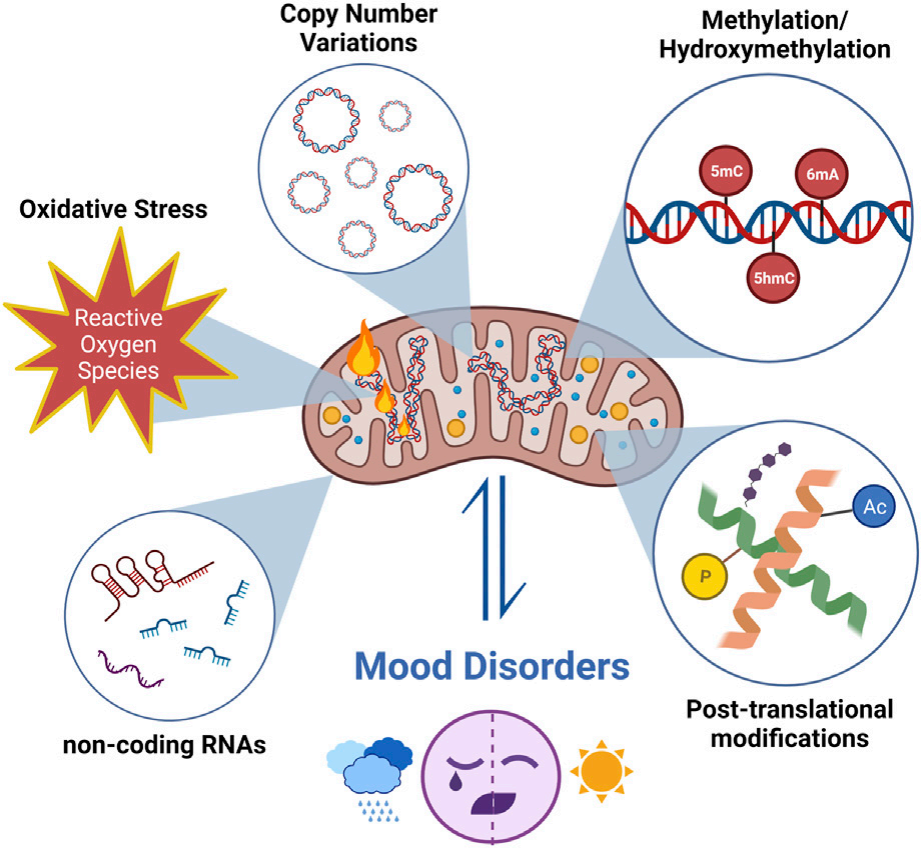
The research about mtDNA modifications focuses on changes in mtDNA copy numbers and oxidation levels, in addition to mitoepigenetic changes in mood disorders. Different types of modifications have been identified for mitoepigenetics. (1) mtDNA methylation/hydroxymethylation; (2) modifications of mitochondrial nucleoids; (3) mitochondrial RNA modifications and modulation of non-coding RNAs (ncRNA) originating from nuclear DNA or mtDNA. (Created with BioRender.com).

Due to methodological limitations, mitochondrial epigenetic changes have not been extensively studied; however, it is known that mtDNA epigenetic changes differ from those in nuclear DNA. Three types of modifications have been identified for mitoepigenetics: 1) mtDNA methylation/hydroxymethylation, 2) modifications of mitochondrial nucleoids, 3) mitochondrial RNA modifications, and modulation of non-coding RNAs (ncRNAs) originating from nuclear DNA or mtDNA (Figure 1).

## mtDNA methylation/hydroxymethylation

The most extensively studied epigenetic mechanism is DNA methylation, particularly in the nuclear DNA. DNA methylation is facilitated by a group of enzymes, known as DNA methyltransferases. These enzymes transfer a methyl group from a co-factor molecule, S-adenosyl-L-methionine, to the C5 position of cytosine and the N6 position of adenine residues, producing 5-methylcytosine (5mC) and N6-methyladenosine (6mA). Both 5mC and 6 mA are involved in either silencing or activating gene transcription. Epigenetic modification of mtDNA has been a topic of controversy and ongoing research. Although it was previously believed that mtDNA did not undergo significant epigenetic modifications, recent studies have challenged this notion (Delsite et al., 2002; Smiraglia et al., 2008; Santos, 2021). The presence of both 5mC and 5-hydroxymethylcytosine (5hmC) alterations in mtDNA suggests that epigenetic modifications may play an important role in mitochondrial functions (Shock et al., 2011; Manev et al., 2012; Bellizzi et al., 2013). A previous study showed that 6 mA in mtDNA can disrupt the DNA binding of mitochondrial transcription factor A (TFAM), thereby reducing mtDNA transcription (Hao et al., 2020). The ten-eleven translocation (TET) protein family of dioxygenases catalyzes an oxidative reaction (hydroxylation) that converts (5mC) to 5-hydroxymethylcytosine (5hmC) in an iron (II)- and α-ketoglutarate-dependent manner (Iyer et al., 2009; Tahiliani et al., 2009; Ito et al., 2010; Dzitoyeva et al., 2012) showed that both 5mC and 5hmC are present in mitochondrial DNA of the mammalian central nervous system (Dzitoyeva et al., 2012). Cytosine methylation is more widespread in eukaryotes and predominantly occurs within CpG dinucleotides located on CpG islands within the cell nucleus (Jin and Robertson, 2013; Lyko, 2018). Methylation of CpG dinucleotides occurs in certain regions, such as heavy strand promoter 1 (HSP1), heavy strand promoter 2 (HSP2), the light strand promoter (LSP), and TFAM binding sites, because mitochondrial DNA contains a low proportion of CpG islands. (Bellizzi et al., 2013; Dostal and Churchill, 2019). MtDNA methylation and hydroxymethylation have been shown that these changes have effects on neurodegenerative diseases such as Alzheimer’s disease, Parkinson’s disease, and amyotrophic lateral sclerosis (Blanch et al., 2016; Stoccoro et al., 2018; Nikolac Perkovic et al., 2021; Stoccoro and Coppedè, 2021; Stoccoro et al., 2022). Several animal studies have shown TET-mediated antidepressant-like effects, but most of them are related to nuclear epigenetic regulation. One study showed that sodium butyrate exerts antidepressant-like effects, with an increase in TET1 leading to elevated 5hmC levels in the BDNF gene in the prefrontal cortex in a rat model of depression (Wei et al., 2014). DNA hydroxymethylase deficiency has been demonstrated lead to a non-negligible epigenetic alteration in response to stress and is implicated in the pathophysiology of depressive behaviors in mice (Cheng et al., 2018). Another study showed that neuroinflammation can decrease 5hmC enrichment in the brain-derived neurotrophic factor (BDNF) gene, which is an essential epigenetic element related to depression-like behaviors (Zhao et al., 2023). In addition, Scola et al. showed that lithium may have a protective effect on rotenone-induced mitochondrial complex I dysfunction and decrease the levels of mtDNA methylation and hydroxymethylation in rat primary cortical neurons (Scola et al., 2014).

mtDNA replication and transcription are impacted by altered methylation in the D-loop, a non-coding region of mtDNA (Shock et al., 2011; Liu et al., 2016). Alterations or mutations in the D-loop region can affect mitochondrial function, contributing to various diseases, such as neurodegenerative disorders and mood disorders (Blanch et al., 2016; Stoccoro et al., 2018; Chung et al., 2019). Blanch et al. reported elevated levels of methylation in the D-loop region of mtDNA in the entorhinal cortex of brain samples from patients with Alzheimer’s disease (Blanch et al., 2016). Stoccoro et al. showed that patients with amyotrophic lateral sclerosis had significantly lower methylation levels in the D-loop region of mtDNA (Stoccoro et al., 2018). A previous study presented that the methylation status of the D-loop region did not differ significantly between individuals with MDD and controls (Chung et al., 2019); however, individuals with MDD displayed increased mtDNA copy number and reduced DNA methylation levels within the PGC1α promoter (Chung et al., 2019). In contrast, Ceylan et al. showed increased methylation in the D-loop region in MDD compared to that in BD and healthy controls (Ceylan et al., 2023).

## Modifications of mitochondrial nucleoids

Nuclear DNA has several post-translational changes of histone proteins (such as methylation, acetylation, phosphorylation, ubiquitination, sumoylation, and poly ADP-ribosylation (parylation)) (Kouzarides, 2007). Unlike nuclear DNA, mitochondrial DNA lacks histones. Mitochondrial nucleoids are distinct entities located within the mitochondria that house mtDNA and associated proteins. These nucleoids play a vital role in the organization and maintenance of the mitochondrial genome and participate in tasks such as mtDNA packaging, maintenance, and the regulation of gene expression (Kukat et al., 2015). Mitochondrial transcription factor A (TFAM), the major component of the mitochondrial nucleoids, mitochondrial single-stranded binding protein, mitochondrial RNA polymerase, and mitochondrial DNA polymerase gamma (POLG) are some of the most well-known nucleoids, and they can be post-translationally modified via acetylation, O-linked glycosylation, and phosphorylation (Sharma et al., 2019). Therefore, epigenetic changes occurring in nucleoid proteins play a crucial role in the regulation of mtDNA gene expression. POLG is responsible for replicating and repairing mitochondrial DNA and is involved in various processes that are critical for maintaining mitochondrial genome integrity. Reportedly, rapid cycling patients with BD exhibit significant downregulation of POLG expression compared with controls, regardless of illness episodes (Munkholm et al., 2015). However, our study group presented unchanged levels of POLG in both euthymic patients and depressive individuals with BD, as well as in individuals with MDD (Yılmaz et al., 2022). Neuron-specific DNA methylation analysis of neurons isolated from certain brain areas that expressed the proofreading-deficient POLG1 transgenic mice showed that mutant mice displayed depression-like and bipolar disorder-like behavioral abnormalities (Kasahara et al., 2016; Sugawara et al., 2022). On the other hand, TFAM, a nuclear-encoded transcription factor, is involved in regulating the replication and transcription of mtDNA, interacts with the DNA to facilitate condensation into nucleoids and binding to the D-loop (Choi et al., 2005), and serves as an instance of a mitochondrial protein subject to post-translational modifications (Suarez et al., 2008; Lu et al., 2013; King et al., 2018). A study showed that mitochondrial poly ADP-ribose polymerase 1 (PARP1) induces NAD + -dependent mitochondrial nucleoid poly ADP-ribosylation, influencing the recruitment of TFAM to stimulate mtDNA transcription and indicating epigenetic nucleoid reorganization (Lee et al., 2022). The expression level of TFAM has been demonstrated to be altered in various cancers, including breast cancer, lung cancer, and melanoma (Fan et al., 2017; Araujo et al., 2018; Lin et al., 2018). However, no data has indicated an association between TFAM and mood disorders. Since mitochondrial epigenetics is a newly recognized research area, no study to date has investigated the posttranslational modifications of nucleoids in mood disorders.

Mitochondrial RNA modifications and modulation of ncRNAs: There are various types of mitochondrial RNAs, including 2 rRNAs, 22 tRNAs, 13 mRNAs, and numerous ncRNAs, found in the mitochondrial matrix. Post-transcriptional mechanisms play a crucial role in governing the translation, maturation, stability, and assembly of mitochondrial RNAs. Modifications of mitochondrial mRNAs, rRNAs, and tRNAs have been linked to various mitochondrial diseases (Dong et al., 2020). N^1^-methyladenosine and N^6^-methyladenosine have been identified as post-transcriptional modifications of mitochondrial RNAs (Wang et al., 2017; Zhang and Jia, 2018). Alterations in the methylation levels of mitochondrial tRNAs have been observed in tumor tissues (Hodgkinson et al., 2014). However, the potential roles of mitochondrial RNA modifications in the pathophysiology of mood disorders have not been elucidated.

RNA transcripts that cannot be translated into proteins are referred to as non-coding RNAs (ncRNAs). ncRNAs, encompassing both small and long ncRNAs, play crucial roles in biological processes such as developmental pathways, cellular responses, and regulatory functions in transcription, and contribute to post-transcriptional processes by regulating gene expression through epigenetic mechanisms. They also play crucial roles in mitochondrial function (Kobayashi et al., 2023). Mitochondria-derived ncRNAs can be found both inside and outside the mitochondria, regulating communication between the mitochondria and nucleus through anterograde and retrograde signals. ncRNAs inside the mitochondria can be transcribed from either the nuclear or mitochondrial genome, and certain mitochondrial ncRNAs are transcribed from the mitochondrial genome before being transported to the cell nuclei or cytosol (Liang et al., 2021). MitomiRs are promising targets for future research in mood disorders.

MicroRNAs (miRNA) are the most abundant ncRNAs and play roles in regulating protein synthesis and controlling gene expression. Numerous studies have provided evidence for the significant role of miRNAs in various psychiatric disorders, including MDD, BD, and schizophrenia (Maffioletti et al., 2016; Cao and Zhen, 2018; Ceylan et al., 2020b; Zhang et al., 2020). MtDNA also encodes miRNAs that are involved in the regulation of mitochondrial gene expression (Sripada et al., 2012). miRNAs localized in mitochondria, whether transported from the nucleus or transcribed in the mitochondria, are known as mitomiRs (Rencelj et al., 2021). Most mitomiRs are encoded in the genome, and a small portion of the mitochondrial genome encodes these mitomiRs (Shinde and Bhadra, 2015). MitomiRs have been demonstrated to be important regulators of mitochondrial function (Rencelj et al., 2021). The regulation of mitochondria by mitomiRs influences the development of numerous diseases caused by mitochondrial dysfunction, contributing to the pathophysiology of conditions such as cardiovascular, metabolic, and neurodegenerative diseases as well as cancers (Dong et al., 2020; Wagner et al., 2022).

Circulating RNAs (circRNAs) are ncRNAs that are involved in depolarization, plasticity, neuronal activity, and synaptic transmission. A recent study showed that 55 circRNAs were altered in individuals with schizophrenia and BD compared to healthy controls, and 71% of these circRNAs were downregulated (Mahmoudi et al., 2021). Mitochondria-encoded circRNAs have also been identified. Four major types of circRNAs have been shown to have an impact on the function of mitochondria, including mecciND1, and mecciND5-mc-COX2-circRNA SCAR (Liu and Shan, 2021).

Long ncRNAs (lncRNAs) are the most abundantly expressed ncRNAs in the brain and contribute to the regulation of chromatin remodeling, protein scaffolding, translation, splicing, and transcription. Studies have confirmed that they interact with epigenetic mediators and transcription factors and subsequently regulate transcription by targeting gene promoters (Qureshi et al., 2010; Kadakkuzha et al., 2015; Quinn and Chang, 2016). Changes in lncRNAs have been associated with depression (Liu et al., 2014), bipolar disorder (Sayad et al., 2019; Shirvani Farsani et al., 2020), schizophrenia (Mishra and Kumar, 2021), autism (Wilkinson and Campbell, 2013), dementia, and other neurodegenerative diseases (Riva et al., 2016). Numerous lncRNA genomic regions have been associated with depression in genome-wide association studies (Zeng et al., 2017), and single-gene polymorphisms in lncRNA genes have been reported to be associated with the risk of depression (Delacrétaz et al., 2015; Ye et al., 2017). Studies have demonstrated the differential regulation of various lncRNA expressions in peripheral blood samples of individuals with MDD or BD compared to healthy controls (Liu et al., 2014; Cui et al., 2016; Cui et al., 2017; Ghafelehbashi et al., 2017; Ye et al., 2017; Naghavi-Gargari et al., 2019; Sayad et al., 2019). In addition, a recent study denoted the increased gene expression levels of *long intergenic ncRNA 173* (LINC00173) in the *postmortem* brain tissue of individuals with BD (Akula et al., 2014).

In recent years, mitochondria-derived ncRNAs have attracted the attention of researchers because of their critical roles in the pathophysiology of many disorders (Zhao et al., 2018; Ren et al., 2023). However, there is currently no published study in the literature that has investigated mitochondrial-encoded ncRNAs, such as miRNAs, circRNAs, and lncRNAs, in mood disorders. Examining the roles of ncRNAs in mitoepigenetics remains a central focus for further exploration.

## Conclusion and future directions

The integration of mitoepigenetics into the broader framework of mood disorder research has the potential to enrich our understanding of the molecular underpinnings of these conditions. Currently, limited research exploring D-loop methylation and mtDNA oxidation in mood disorders suggests the need for a more detailed focus on this area of research. In addition, investigating post-translational modifications of nucleoids and their impact on mitochondrial function in mood disorders could provide crucial insights into the epigenetic regulation of mtDNA. Mitochondrial RNA, including ncRNAs such as mitomiRs, circRNAs, and lncRNAs, emerges as a dynamic component influencing mitochondrial and nuclear interactions. miRNAs, in particular, have been implicated in various psychiatric disorders, and mitochondrial-encoded mitomiRs present a novel dimension in understanding mitochondrial contributions to mood regulation. Exploring the roles of mitomiRs, circRNAs, and lncRNAs in mood disorders can deepen our understanding of the intricate regulatory networks governing the mitochondrial contributions to these conditions. Considering the multifactorial nature of mood disorders, future research should explore the interactions between mitoepigenetics and environmental factors such as stressors, trauma, and lifestyle to comprehensively understand the etiology of these conditions. Translating the insights gained from mitochondrial epigenetic research into clinical applications holds great promise. Identifying therapeutic targets related to mitoepigenetics could pave the way for innovative interventions in mood disorders.

## References

[B1] Ahmadimanesh M. Abbaszadegan M. R. Morshedi Rad D. Moallem S. A. Mohammadpour A. H. Ghahremani M. H. (2019). Effects of selective serotonin reuptake inhibitors on DNA damage in patients with depression. J. Psychopharmacol. 33, 1364–1376. 10.1177/0269881119874461 31556787

[B2] Akula N. Barb J. Jiang X. Wendland J. R. Choi K. H. Sen S. K. (2014). RNA-sequencing of the brain transcriptome implicates dysregulation of neuroplasticity, circadian rhythms and GTPase binding in bipolar disorder. Mol. Psychiatry 19, 1179–1185. 10.1038/mp.2013.170 24393808 PMC5560442

[B3] Allen J. Romay-Tallon R. Brymer K. J. Caruncho H. J. Kalynchuk L. E. (2018). Mitochondria and mood: mitochondrial dysfunction as a key player in the manifestation of depression. Front. Neurosci. 12, 386. 10.3389/fnins.2018.00386 29928190 PMC5997778

[B4] Araujo L. F. Siena A. D. D. Plaça J. R. Brotto D. B. Barros II Muys B. R. (2018). Mitochondrial transcription factor A (TFAM) shapes metabolic and invasion gene signatures in melanoma. Sci. Rep. 8, 14190. 10.1038/s41598-018-31170-6 30242167 PMC6155108

[B5] Bansal Y. Kuhad A. (2016). Mitochondrial dysfunction in depression. Curr. Neuropharmacol. 14, 610–618. 10.2174/1570159x14666160229114755 26923778 PMC4981740

[B6] Bellizzi D. D'Aquila P. Scafone T. Giordano M. Riso V. Riccio A. (2013). The control region of mitochondrial DNA shows an unusual CpG and non-CpG methylation pattern. DNA Res. 20, 537–547. 10.1093/dnares/dst029 23804556 PMC3859322

[B7] Blanch M. Mosquera J. L. Ansoleaga B. Ferrer I. Barrachina M. (2016). Altered mitochondrial DNA methylation pattern in alzheimer disease-related pathology and in Parkinson disease. Am. J. Pathol. 186, 385–397. 10.1016/j.ajpath.2015.10.004 26776077

[B8] Bock F. J. Tait S. W. G. (2020). Mitochondria as multifaceted regulators of cell death. Nat. Rev. Mol. Cell Biol. 21, 85–100. 10.1038/s41580-019-0173-8 31636403

[B9] Bodenstein D. F. Kim H. K. Brown N. C. Navaid B. Young L. T. Andreazza A. C. (2019). Mitochondrial DNA content and oxidation in bipolar disorder and its role across brain regions. NPJ Schizophr. 5, 21. 10.1038/s41537-019-0089-5 31797868 PMC6892804

[B10] Cai N. Chang S. Li Y. Li Q. Hu J. Liang J. (2015). Molecular signatures of major depression. Curr. Biol. 25, 1146–1156. 10.1016/j.cub.2015.03.008 25913401 PMC4425463

[B11] Cao T. Zhen X. C. (2018). Dysregulation of miRNA and its potential therapeutic application in schizophrenia. CNS Neurosci. Ther. 24, 586–597. 10.1111/cns.12840 29529357 PMC6490029

[B12] Cavalcante G. C. Magalhães L. Ribeiro-Dos-Santos Â. Vidal A. F. (2020). Mitochondrial epigenetics: non-coding RNAs as a novel layer of complexity. Int. J. Mol. Sci. 21, 1838. 10.3390/ijms21051838 32155913 PMC7084767

[B13] Çelik H. Tuna G. Ceylan D. Küçükgöncü S. (2023). A comparative meta-analysis of peripheral 8-hydroxy-2'-deoxyguanosine (8-OHdG) or 8-oxo-7,8-dihydro-2'-deoxyguanosine (8-oxo-dG) levels across mood episodes in bipolar disorder. Psychoneuroendocrinology 151, 106078. 10.1016/j.psyneuen.2023.106078 36931055

[B14] Ceylan D. Karaçiçek B. Tufekci K. U. Aksahin I. C. Hun Şenol Ş. Genc S. (2023). Mitochondrial DNA oxidation, methylation, and copy number alterations in major and bipolar depression. Front. Psychiatry 14, 1304660. 10.3389/fpsyt.2023.1304660 38161720 PMC10755902

[B15] Ceylan D. Tufekci K. U. Keskinoglu P. Genc S. Özerdem A. (2020a). Circulating exosomal microRNAs in bipolar disorder. J. Affect Disord. 262, 99–107. 10.1016/j.jad.2019.10.038 31726266

[B16] Ceylan D. Tuna G. Kirkali G. Tunca Z. Can G. Arat H. E. (2018). Oxidatively-induced DNA damage and base excision repair in euthymic patients with bipolar disorder. DNA Repair (Amst) 65, 64–72. 10.1016/j.dnarep.2018.03.006 29626765 PMC7243967

[B17] Ceylan D. Yılmaz S. Tuna G. Kant M. Er A. Ildız A. (2020b). Alterations in levels of 8-Oxo-2'-deoxyguanosine and 8-Oxoguanine DNA glycosylase 1 during a current episode and after remission in unipolar and bipolar depression. Psychoneuroendocrinology 114, 104600. 10.1016/j.psyneuen.2020.104600 32062372

[B18] Chang C. C. Jou S. H. Lin T. T. Lai T. J. Liu C. S. (2015). Mitochondria DNA change and oxidative damage in clinically stable patients with major depressive disorder. PLoS One 10, e0125855. 10.1371/journal.pone.0125855 25946463 PMC4422713

[B19] Cheng Y. Sun M. Chen L. Li Y. Lin L. Yao B. (2018). Ten-eleven translocation proteins modulate the response to environmental stress in mice. Cell Rep. 25, 3194–3203. 10.1016/j.celrep.2018.11.061 30540950 PMC6350936

[B20] Choi Y. S. Ryu B. K. Min H. K. Lee S. W. Pak Y. K. (2005). Analysis of proteome bound to D-loop region of mitochondrial DNA by DNA-linked affinity chromatography and reverse-phase liquid chromatography/tandem mass spectrometry. Ann. N. Y. Acad. Sci. 1042, 88–100. 10.1196/annals.1338.009 15965050

[B21] Chung J. K. Ahn Y. M. Kim S. A. Joo E. J. (2022). Differences in mitochondrial DNA copy number between patients with bipolar I and II disorders. J. Psychiatr. Res. 145, 325–333. 10.1016/j.jpsychires.2020.11.016 33190840

[B22] Chung J. K. Lee S. Y. Park M. Joo E. J. Kim S. A. (2019). Investigation of mitochondrial DNA copy number in patients with major depressive disorder. Psychiatry Res. 282, 112616. 10.1016/j.psychres.2019.112616 31639552

[B23] Cui X. Niu W. Kong L. He M. Jiang K. Chen S. (2017). Long noncoding RNA expression in peripheral blood mononuclear cells and suicide risk in Chinese patients with major depressive disorder. Brain Behav. 7, e00711. 10.1002/brb3.711 28638716 PMC5474714

[B24] Cui X. Sun X. Niu W. Kong L. He M. Zhong A. (2016). Long non-coding RNA: potential diagnostic and therapeutic biomarker for major depressive disorder. Med. Sci. Monit. 22, 5240–5248. 10.12659/msm.899372 28039689 PMC5221417

[B25] Culmsee C. Michels S. Scheu S. Arolt V. Dannlowski U. Alferink J. (2018). Mitochondria, microglia, and the immune system-how are they linked in affective disorders? Front. Psychiatry 9, 739. 10.3389/fpsyt.2018.00739 30687139 PMC6333629

[B26] Czarny P. Bialek K. Ziolkowska S. Strycharz J. Sliwinski T. (2020a). DNA damage and repair in neuropsychiatric disorders. What do we know and what are the future perspectives? Mutagenesis 35, 79–106. 10.1093/mutage/gez035 31676908

[B27] Czarny P. Wigner P. Strycharz J. Swiderska E. Synowiec E. Szatkowska M. (2020b). Mitochondrial DNA copy number, damage, repair and degradation in depressive disorder. World J. Biol. Psychiatry 21, 91–101. 10.1080/15622975.2019.1588993 31081430

[B28] Das S. C. Hjelm B. E. Rollins B. L. Sequeira A. Morgan L. Omidsalar A. A. (2022). Mitochondria DNA copy number, mitochondria DNA total somatic deletions, Complex I activity, synapse number, and synaptic mitochondria number are altered in schizophrenia and bipolar disorder. Transl. Psychiatry 12, 353. 10.1038/s41398-022-02127-1 36042222 PMC9427957

[B29] Delacrétaz A. Preisig M. Vandenberghe F. Saigi Morgui N. Quteineh L. Choong E. (2015). Influence of MCHR2 and MCHR2-AS1 genetic polymorphisms on body mass index in psychiatric patients and in population-based subjects with present or past atypical depression. PLoS One 10, e0139155. 10.1371/journal.pone.0139155 26461262 PMC4604197

[B30] Delsite R. Kachhap S. Anbazhagan R. Gabrielson E. Singh K. K. (2002). Nuclear genes involved in mitochondria-to-nucleus communication in breast cancer cells. Mol. Cancer 1, 6. 10.1186/1476-4598-1-6 12495447 PMC149409

[B31] Dong Z. Pu L. Cui H. (2020). Mitoepigenetics and its emerging roles in cancer. Front. Cell Dev. Biol. 8, 4. 10.3389/fcell.2020.00004 32039210 PMC6989428

[B32] Dostal V. Churchill M. E. A. (2019). Cytosine methylation of mitochondrial DNA at CpG sequences impacts transcription factor A DNA binding and transcription. Biochim. Biophys. Acta Gene Regul. Mech. 1862, 598–607. 10.1016/j.bbagrm.2019.01.006 30807854 PMC7806247

[B33] Dzitoyeva S. Chen H. Manev H. (2012). Effect of aging on 5-hydroxymethylcytosine in brain mitochondria. Neurobiol. Aging 33, 2881–2891. 10.1016/j.neurobiolaging.2012.02.006 22445327 PMC3462297

[B34] Fan X. Zhou S. Zheng M. Deng X. Yi Y. Huang T. (2017). MiR-199a-3p enhances breast cancer cell sensitivity to cisplatin by downregulating TFAM (TFAM). Biomed. Pharmacother. 88, 507–514. 10.1016/j.biopha.2017.01.058 28126676

[B35] Föcking M. Dicker P. Lopez L. M. Hryniewiecka M. Wynne K. English J. A. (2016). Proteomic analysis of the postsynaptic density implicates synaptic function and energy pathways in bipolar disorder. Transl. Psychiatry 6, e959. 10.1038/tp.2016.224 27898073 PMC5290351

[B36] Fries G. R. Bauer I. E. Scaini G. Wu M. J. Kazimi I. F. Valvassori S. S. (2017). Accelerated epigenetic aging and mitochondrial DNA copy number in bipolar disorder. Transl. Psychiatry 7, 1283. 10.1038/s41398-017-0048-8 29225347 PMC5802567

[B37] Ghafelehbashi H. Pahlevan Kakhki M. Kular L. Moghbelinejad S. Ghafelehbashi S. H. (2017). Decreased expression of IFNG-AS1, IFNG and IL-1B inflammatory genes in medicated schizophrenia and bipolar patients. Scand. J. Immunol. 86, 479–485. 10.1111/sji.12620 29032575

[B38] Ghosh S. Singh K. K. Sengupta S. Scaria V. (2015). Mitoepigenetics: the different shades of grey. Mitochondrion 25, 60–66. 10.1016/j.mito.2015.09.003 26437363

[B39] Hao Z. Wu T. Cui X. Zhu P. Tan C. Dou X. (2020). N(6)-Deoxyadenosine methylation in mammalian mitochondrial DNA. Mol. Cell 78, 382–395. 10.1016/j.molcel.2020.02.018 32183942 PMC7214128

[B40] Hodgkinson A. Idaghdour Y. Gbeha E. Grenier J. C. Hip-Ki E. Bruat V. (2014). High-resolution genomic analysis of human mitochondrial RNA sequence variation. Science 344, 413–415. 10.1126/science.1251110 24763589

[B41] Ito S. D'Alessio A. C. Taranova O. V. Hong K. Sowers L. C. Zhang Y. (2010). Role of Tet proteins in 5mC to 5hmC conversion, ES-cell self-renewal and inner cell mass specification. Nature 466, 1129–1133. 10.1038/nature09303 20639862 PMC3491567

[B42] Iyer L. M. Tahiliani M. Rao A. Aravind L. (2009). Prediction of novel families of enzymes involved in oxidative and other complex modifications of bases in nucleic acids. Cell Cycle 8, 1698–1710. 10.4161/cc.8.11.8580 19411852 PMC2995806

[B43] Jin B. Robertson K. D. (2013). DNA methyltransferases, DNA damage repair, and cancer. Adv. Exp. Med. Biol. 754, 3–29. 10.1007/978-1-4419-9967-2_1 22956494 PMC3707278

[B44] Kadakkuzha B. M. Liu X. A. McCrate J. Shankar G. Rizzo V. Afinogenova A. (2015). Transcriptome analyses of adult mouse brain reveal enrichment of lncRNAs in specific brain regions and neuronal populations. Front. Cell Neurosci. 9, 63. 10.3389/fncel.2015.00063 25798087 PMC4351618

[B45] Kakiuchi C. Ishiwata M. Kametani M. Nelson C. Iwamoto K. Kato T. (2005). Quantitative analysis of mitochondrial DNA deletions in the brains of patients with bipolar disorder and schizophrenia. Int. J. Neuropsychopharmacol. 8, 515–522. 10.1017/s1461145705005213 16202181

[B46] Karabatsiakis A. Böck C. Salinas-Manrique J. Kolassa S. Calzia E. Dietrich D. E. (2014). Mitochondrial respiration in peripheral blood mononuclear cells correlates with depressive subsymptoms and severity of major depression. Transl. Psychiatry 4, e397. 10.1038/tp.2014.44 PMC4080325 26126180

[B47] Kasahara T. Kato T. (2018). What can mitochondrial DNA analysis tell us about mood disorders? Biol. Psychiatry 83, 731–738. 10.1016/j.biopsych.2017.09.010 29102411

[B48] Kasahara T. Takata A. Kato T. M. Kubota-Sakashita M. Sawada T. Kakita A. (2016). Depression-like episodes in mice harboring mtDNA deletions in paraventricular thalamus. Mol. Psychiatry 21, 39–48. 10.1038/mp.2015.156 26481320 PMC5414076

[B49] Kim M. Y. Lee J. W. Kang H. C. Kim E. Lee D. C. (2011). Leukocyte mitochondrial DNA (mtDNA) content is associated with depression in old women. Arch. Gerontol. Geriatr. 53, e218–e221. 10.1016/j.archger.2010.11.019 21159390

[B50] King G. A. Hashemi Shabestari M. Taris K. H. Pandey A. K. Venkatesh S. Thilagavathi J. (2018). Acetylation and phosphorylation of human TFAM regulate TFAM-DNA interactions via contrasting mechanisms. Nucleic Acids Res. 46, 3633–3642. 10.1093/nar/gky204 29897602 PMC5909435

[B51] Kobayashi A. Takeiwa T. Ikeda K. Inoue S. (2023). Roles of noncoding RNAs in regulation of mitochondrial electron transport chain and oxidative phosphorylation. Int. J. Mol. Sci. 24, 9414. 10.3390/ijms24119414 37298366 PMC10253563

[B52] Kolar D. Kleteckova L. Brozka H. Vales K. (2021). Mini-review: brain energy metabolism and its role in animal models of depression, bipolar disorder, schizophrenia and autism. Neurosci. Lett. 760, 136003. 10.1016/j.neulet.2021.136003 34098028

[B53] Konttinen H. Cabral-da-Silva M. E. C. Ohtonen S. Wojciechowski S. Shakirzyanova A. Caligola S. (2019). PSEN1ΔE9, APPswe, and APOE4 confer disparate phenotypes in human iPSC-derived microglia. Stem Cell Rep. 13, 669–683. 10.1016/j.stemcr.2019.08.004 PMC6829767 31522977

[B54] Kouzarides T. (2007). Chromatin modifications and their function. Cell 128, 693–705. 10.1016/j.cell.2007.02.005 17320507

[B55] Kucuker M. U. Ozerdem A. Ceylan D. Cabello-Arreola A. Ho A. M. C. Joseph B. (2022). The role of base excision repair in major depressive disorder and bipolar disorder. J. Affect Disord. 306, 288–300. 10.1016/j.jad.2022.03.033 35306122

[B56] Kuffner K. Triebelhorn J. Meindl K. Benner C. Manook A. Sudria-Lopez D. (2020). Major depressive disorder is associated with impaired mitochondrial function in skin fibroblasts. Cells 9, 884. 10.3390/cells9040884 32260327 PMC7226727

[B57] Kukat C. Davies K. M. Wurm C. A. Spåhr H. Bonekamp N. A. Kühl I. (2015). Cross-strand binding of TFAM to a single mtDNA molecule forms the mitochondrial nucleoid. Proc. Natl. Acad. Sci. U. S. A. 112, 11288–11293. 10.1073/pnas.1512131112 26305956 PMC4568684

[B58] Lapp H. E. Bartlett A. A. Hunter R. G. (2019). Stress and glucocorticoid receptor regulation of mitochondrial gene expression. J. Mol. Endocrinol. 62, R121–R128. 10.1530/jme-18-0152 30082335

[B59] Lee J. H. Hussain M. Kim E. W. Cheng S. J. Leung A. K. L. Fakouri N. B. (2022). Mitochondrial PARP1 regulates NAD(+)-dependent poly ADP-ribosylation of mitochondrial nucleoids. Exp. Mol. Med. 54, 2135–2147. 10.1038/s12276-022-00894-x 36473936 PMC9794712

[B60] Legrand A. Iftimovici A. Khayachi A. Chaumette B. (2021). Epigenetics in bipolar disorder: a critical review of the literature. Psychiatr. Genet. 31, 1–12. 10.1097/ypg.0000000000000267 33290382

[B61] Liang H. Liu J. Su S. Zhao Q. (2021). Mitochondrial noncoding RNAs: new wine in an old bottle. RNA Biol. 18, 2168–2182. 10.1080/15476286.2021.1935572 34110970 PMC8632133

[B62] Lin C. S. Liu L. T. Ou L. H. Pan S. C. Lin C. I. Wei Y. H. (2018). Role of mitochondrial function in the invasiveness of human colon cancer cells. Oncol. Rep. 39, 316–330. 10.3892/or.2017.6087 29138850

[B63] Lindqvist D. Wolkowitz O. M. Picard M. Ohlsson L. Bersani F. S. Fernström J. (2018). Circulating cell-free mitochondrial DNA, but not leukocyte mitochondrial DNA copy number, is elevated in major depressive disorder. Neuropsychopharmacology 43, 1557–1564. 10.1038/s41386-017-0001-9 29453441 PMC5983469

[B64] Liu B. Du Q. Chen L. Fu G. Li S. Fu L. (2016). CpG methylation patterns of human mitochondrial DNA. Sci. Rep. 6, 23421. 10.1038/srep23421 26996456 PMC4800444

[B65] Liu X. Shan G. (2021). Mitochondria encoded non-coding RNAs in cell Physiology. Front. Cell Dev. Biol. 9, 713729. 10.3389/fcell.2021.713729 34395442 PMC8362354

[B66] Liu Z. Li X. Sun N. Xu Y. Meng Y. Yang C. (2014). Microarray profiling and co-expression network analysis of circulating lncRNAs and mRNAs associated with major depressive disorder. PLoS One 9, e93388. 10.1371/journal.pone.0093388 24676134 PMC3968145

[B67] Lu B. Lee J. Nie X. Li M. Morozov Y. I. Venkatesh S. (2013). Phosphorylation of human TFAM in mitochondria impairs DNA binding and promotes degradation by the AAA+ Lon protease. Mol. Cell 49, 121–132. 10.1016/j.molcel.2012.10.023 23201127 PMC3586414

[B68] Lundberg M. Millischer V. Backlund L. Martinsson L. Stenvinkel P. Sellgren C. M. (2020). Lithium and the interplay between telomeres and mitochondria in bipolar disorder. Front. Psychiatry 11, 586083. 10.3389/fpsyt.2020.586083 33132941 PMC7553080

[B69] Lyko F. (2018). The DNA methyltransferase family: a versatile toolkit for epigenetic regulation. Nat. Rev. Genet. 19, 81–92. 10.1038/nrg.2017.80 29033456

[B70] Maffioletti E. Cattaneo A. Rosso G. Maina G. Maj C. Gennarelli M. (2016). Peripheral whole blood microRNA alterations in major depression and bipolar disorder. J. Affect Disord. 200, 250–258. 10.1016/j.jad.2016.04.021 27152760

[B71] Mahmoudi E. Green M. J. Cairns M. J. (2021). Dysregulation of circRNA expression in the peripheral blood of individuals with schizophrenia and bipolar disorder. J. Mol. Med. Berl. 99, 981–991. 10.1007/s00109-021-02070-6 33782720

[B72] Malhi G. S. Mann J. J. (2018). Depression. Lancet 392, 2299–2312. 10.1016/s0140-6736(18)31948-2 30396512

[B73] Manev H. (2014). Mitoepigenetics and neuropsychiatric disorders. Epigenetics Psychiatry 2014, 463–478. 10.1016/B978-0-12-417114-5.00022-X

[B74] Manev H. Dzitoyeva S. Chen H. (2012). Mitochondrial DNA: a blind spot in neuroepigenetics. Biomol. Concepts 3, 107–115. 10.1515/bmc-2011-0058 22639700 PMC3359012

[B75] Manji H. Kato T. Di Prospero N. A. Ness S. Beal M. F. Krams M. (2012). Impaired mitochondrial function in psychiatric disorders. Nat. Rev. Neurosci. 13, 293–307. 10.1038/nrn3229 22510887

[B76] Manoli I. Alesci S. Blackman M. R. Su Y. A. Rennert O. M. Chrousos G. P. (2007). Mitochondria as key components of the stress response. Trends Endocrinol. Metab. 18, 190–198. 10.1016/j.tem.2007.04.004 17500006

[B77] Menke A. Klengel T. Binder E. B. (2012). Epigenetics, depression and antidepressant treatment. Curr. Pharm. Des. 18, 5879–5889. 10.2174/138161212803523590 22681167

[B78] Midzak A. Papadopoulos V. (2016). Adrenal mitochondria and steroidogenesis: from individual proteins to functional protein assemblies. Front. Endocrinol. (Lausanne) 7, 106. 10.3389/fendo.2016.00106 27524977 PMC4965458

[B79] Mikhed Y. Görlach A. Knaus U. G. Daiber A. (2015). Redox regulation of genome stability by effects on gene expression, epigenetic pathways and DNA damage/repair. Redox Biol. 5, 275–289. 10.1016/j.redox.2015.05.008 26079210 PMC4475862

[B80] Miller W. L. (2011). Role of mitochondria in steroidogenesis. Endocr. Dev. 20, 1–19. 10.1159/000321204 21164254

[B81] Mishra P. Kumar S. (2021). Association of lncRNA with regulatory molecular factors in brain and their role in the pathophysiology of schizophrenia. Metab. Brain Dis. 36, 849–858. 10.1007/s11011-021-00692-w 33608830

[B82] Morava E. Kozicz T. (2013). Mitochondria and the economy of stress (mal)adaptation. Neurosci. Biobehav Rev. 37, 668–680. 10.1016/j.neubiorev.2013.02.005 23415702

[B83] Munkholm K. Peijs L. Vinberg M. Kessing L. V. (2015). A composite peripheral blood gene expression measure as a potential diagnostic biomarker in bipolar disorder. Transl. Psychiatry 5, e614. 10.1038/tp.2015.110 26241352 PMC4564565

[B84] Naghavi-Gargari B. Zahirodin A. Ghaderian S. M. H. Shirvani-Farsani Z. (2019). Significant increasing of DISC2 long non-coding RNA expression as a potential biomarker in bipolar disorder. Neurosci. Lett. 696, 206–211. 10.1016/j.neulet.2018.12.044 30599263

[B85] Nicod J. Wagner S. Vonberg F. Bhomra A. Schlicht K. F. Tadic A. (2016). The amount of mitochondrial DNA in blood reflects the course of a depressive episode. Biol. Psychiatry 80, e41–e42. 10.1016/j.biopsych.2015.12.019 26917357

[B86] Nikolac Perkovic M. Videtic Paska A. Konjevod M. Kouter K. Svob Strac D. Nedic Erjavec G. (2021). Epigenetics of Alzheimer's disease. Biomolecules 11, 195. 10.3390/biom11020195 33573255 PMC7911414

[B87] Penner-Goeke S. Binder E. B. (2019). Epigenetics and depression *dialogues clin neurosci* . Dialogues Clin. Neurosci. 21, 397–405. 10.31887/DCNS.2019.21.4/ebinder 31949407 PMC6952745

[B88] Quinn J. J. Chang H. Y. (2016). Unique features of long non-coding RNA biogenesis and function. Nat. Rev. Genet. 17, 47–62. 10.1038/nrg.2015.10 26666209

[B89] Qureshi I. A. Mattick J. S. Mehler M. F. (2010). Long non-coding RNAs in nervous system function and disease. Brain Res. 1338, 20–35. 10.1016/j.brainres.2010.03.110 20380817 PMC2883659

[B90] Ren B. Guan M. X. Zhou T. Cai X. Shan G. (2023). Emerging functions of mitochondria-encoded noncoding RNAs. Trends Genet. 39, 125–139. 10.1016/j.tig.2022.08.004 36137834

[B91] Rencelj A. Gvozdenovic N. Cemazar M. (2021). MitomiRs: their roles in mitochondria and importance in cancer cell metabolism. Radiol. Oncol. 55, 379–392. 10.2478/raon-2021-0042 34821131 PMC8647792

[B92] Riva P. Ratti A. Venturin M. (2016). The long non-coding RNAs in neurodegenerative diseases: novel mechanisms of pathogenesis. Curr. Alzheimer Res. 13, 1219–1231. 10.2174/1567205013666160622112234 27338628

[B93] Rizzuto R. De Stefani D. Raffaello A. Mammucari C. (2012). Mitochondria as sensors and regulators of calcium signalling. Nat. Rev. Mol. Cell Biol. 13, 566–578. 10.1038/nrm3412 22850819

[B94] Saavedra K. Molina-Márquez A. M. Saavedra N. Zambrano T. Salazar L. A. (2016). Epigenetic modifications of major depressive disorder. Int. J. Mol. Sci. 17, 1279. 10.3390/ijms17081279 27527165 PMC5000676

[B95] Santos J. H. (2021). Mitochondria signaling to the epigenome: a novel role for an old organelle. Free Radic. Biol. Med. 170, 59–69. 10.1016/j.freeradbiomed.2020.11.016 33271282 PMC8166959

[B96] Sayad A. Taheri M. Omrani M. D. Fallah H. Kholghi Oskooei V. Ghafouri-Fard S. (2019). Peripheral expression of long non-coding RNAs in bipolar patients. J. Affect Disord. 249, 169–174. 10.1016/j.jad.2019.02.034 30772744

[B97] Scola G. Kim H. K. Young L. T. Salvador M. Andreazza A. C. (2014). Lithium reduces the effects of rotenone-induced complex I dysfunction on DNA methylation and hydroxymethylation in rat cortical primary neurons. Psychopharmacol. Berl. 231, 4189–4198. 10.1007/s00213-014-3565-7 24777143

[B98] Sharma N. Pasala M. S. Prakash A. (2019). Mitochondrial DNA: epigenetics and environment. Environ. Mol. Mutagen 60, 668–682. 10.1002/em.22319 31335990 PMC6941438

[B99] Shinde S. Bhadra U. (2015). A complex genome-microRNA interplay in human mitochondria. Biomed. Res. Int. 2015, 206382. 10.1155/2015/206382 25695052 PMC4324738

[B100] Shirvani Farsani Z. Zahirodin A. Ghaderian S. M. H. Shams J. Naghavi Gargari B. (2020). The role of long non-coding RNA MALAT1 in patients with bipolar disorder. Metab. Brain Dis. 35, 1077–1083. 10.1007/s11011-020-00580-9 32458337

[B101] Shock L. S. Thakkar P. V. Peterson E. J. Moran R. G. Taylor S. M. (2011). DNA methyltransferase 1, cytosine methylation, and cytosine hydroxymethylation in mammalian mitochondria. Proc. Natl. Acad. Sci. U. S. A. 108, 3630–3635. 10.1073/pnas.1012311108 21321201 PMC3048134

[B102] Smiraglia D. J. Kulawiec M. Bistulfi G. L. Gupta S. G. Singh K. K. (2008). A novel role for mitochondria in regulating epigenetic modification in the nucleus. Cancer Biol. Ther. 7, 1182–1190. 10.4161/cbt.7.8.6215 18458531 PMC2639623

[B103] Sripada L. Tomar D. Prajapati P. Singh R. Singh A. K. Singh R. (2012). Systematic analysis of small RNAs associated with human mitochondria by deep sequencing: detailed analysis of mitochondrial associated miRNA. PLoS One 7, e44873. 10.1371/journal.pone.0044873 22984580 PMC3439422

[B104] Stoccoro A. Baldacci F. Ceravolo R. Giampietri L. Tognoni G. Siciliano G. (2022). Increase in mitochondrial D-loop region methylation levels in mild cognitive impairment individuals. Int. J. Mol. Sci. 23, 5393. 10.3390/ijms23105393 35628202 PMC9142993

[B105] Stoccoro A. Coppedè F. (2021). Mitochondrial DNA methylation and human diseases. Int. J. Mol. Sci. 22, 4594. 10.3390/ijms22094594 33925624 PMC8123858

[B106] Stoccoro A. Mosca L. Carnicelli V. Cavallari U. Lunetta C. Marocchi A. (2018). Mitochondrial DNA copy number and D-loop region methylation in carriers of amyotrophic lateral sclerosis gene mutations. Epigenomics 10, 1431–1443. 10.2217/epi-2018-0072 30088417

[B107] Stork C. Renshaw P. F. (2005). Mitochondrial dysfunction in bipolar disorder: evidence from magnetic resonance spectroscopy research. Mol. Psychiatry 10, 900–919. 10.1038/sj.mp.4001711 16027739

[B108] Suarez J. Hu Y. Makino A. Fricovsky E. Wang H. Dillmann W. H. (2008). Alterations in mitochondrial function and cytosolic calcium induced by hyperglycemia are restored by mitochondrial transcription factor A in cardiomyocytes. Am. J. Physiol. Cell Physiol. 295, C1561–C1568. 10.1152/ajpcell.00076.2008 19060297 PMC2603561

[B109] Sugawara H. Bundo M. Kasahara T. Nakachi Y. Ueda J. Kubota-Sakashita M. (2022). Cell-type-specific DNA methylation analysis of the frontal cortices of mutant Polg1 transgenic mice with neuronal accumulation of deleted mitochondrial DNA. Mol. Brain 15, 9. 10.1186/s13041-021-00894-4 34991677 PMC8740475

[B110] Sullivan P. F. Neale M. C. Kendler K. S. (2000). Genetic epidemiology of major depression: review and meta-analysis. Am. J. Psychiatry 157, 1552–1562. 10.1176/appi.ajp.157.10.1552 11007705

[B111] Tahiliani M. Koh K. P. Shen Y. Pastor W. A. Bandukwala H. Brudno Y. (2009). Conversion of 5-methylcytosine to 5-hydroxymethylcytosine in mammalian DNA by MLL partner TET1. Science 324, 930–935. 10.1126/science.1170116 19372391 PMC2715015

[B112] Tsujii N. Otsuka I. Okazaki S. Yanagi M. Numata S. Yamaki N. (2019). Mitochondrial DNA copy number raises the potential of left frontopolar hemodynamic response as a diagnostic marker for distinguishing bipolar disorder from major depressive disorder. Front. Psychiatry 10, 312. 10.3389/fpsyt.2019.00312 31139101 PMC6518968

[B113] Wagner A. Kosnacova H. Chovanec M. Jurkovicova D. (2022). Mitochondrial genetic and epigenetic regulations in cancer: therapeutic potential. Int. J. Mol. Sci. 23, 7897. 10.3390/ijms23147897 35887244 PMC9321253

[B114] Wang D. Li Z. Liu W. Zhou J. Ma X. Tang J. (2018). Differential mitochondrial DNA copy number in three mood states of bipolar disorder. BMC Psychiatry 18, 149. 10.1186/s12888-018-1717-8 29801445 PMC5970444

[B115] Wang Z. Tang K. Zhang D. Wan Y. Wen Y. Lu Q. (2017). High-throughput m6A-seq reveals RNA m6A methylation patterns in the chloroplast and mitochondria transcriptomes of *Arabidopsis thaliana* . PLoS One 12, e0185612. 10.1371/journal.pone.0185612 29131848 PMC5683568

[B116] Wei Y. Melas P. A. Wegener G. Mathé A. A. Lavebratt C. (2014). Antidepressant-like effect of sodium butyrate is associated with an increase in TET1 and in 5-hydroxymethylation levels in the Bdnf gene. Int. J. Neuropsychopharmacol. 18, pyu032. 10.1093/ijnp/pyu032 25618518 PMC4368891

[B117] Wilkinson B. Campbell D. B. (2013). Contribution of long noncoding RNAs to autism spectrum disorder risk. Int. Rev. Neurobiol. 113, 35–59. 10.1016/b978-0-12-418700-9.00002-2 24290382

[B118] Wong-Riley M. T. Tripathi S. C. Trusk T. C. Hoppe D. A. (1989). Effect of retinal impulse blockage on cytochrome oxidase-rich zones in the macaque striate cortex: I. Quantitative electron-microscopic (EM) analysis of neurons. Vis. Neurosci. 2, 483–497. 10.1017/s0952523800012384 2562109

[B119] Xian H. Karin M. (2023). Oxidized mitochondrial DNA: a protective signal gone awry. Trends Immunol. 44, 188–200. 10.1016/j.it.2023.01.006 36739208 PMC12045651

[B120] Yakes F. M. Van Houten B. (1997). Mitochondrial DNA damage is more extensive and persists longer than nuclear DNA damage in human cells following oxidative stress. Proc. Natl. Acad. Sci. U. S. A. 94, 514–519. 10.1073/pnas.94.2.514 9012815 PMC19544

[B121] Ye N. Rao S. Du T. Hu H. Liu Z. Shen Y. (2017). Intergenic variants may predispose to major depression disorder through regulation of long non-coding RNA expression. Gene 601, 21–26. 10.1016/j.gene.2016.11.041 27940106

[B122] Yılmaz S. Akan P. Baysal K. Özerdem A. Ceylan D. (2022). DNA base excision repair genes in unipolar and bipolar depression. Neurosci. Appl. 1, 69. 10.1016/j.nsa.2022.100183

[B123] Zeng Y. Lin R. Liu L. Liu Y. Li Y. (2019). Ambient air pollution exposure and risk of depression: a systematic review and meta-analysis of observational studies. Psychiatry Res. 276, 69–78. 10.1016/j.psychres.2019.04.019 31029037

[B124] Zeng Y. Navarro P. Shirali M. Howard D. M. Adams M. J. Hall L. S. (2017). Genome-wide regional heritability mapping identifies a locus within the TOX2 gene associated with major depressive disorder. Biol. Psychiatry 82, 312–321. 10.1016/j.biopsych.2016.12.012 28153336 PMC5553996

[B125] Zhang C. Jia G. (2018). Reversible RNA modification N(1)-methyladenosine (m(1)A) in mRNA and tRNA. Genomics Proteomics Bioinforma. 16, 155–161. 10.1016/j.gpb.2018.03.003 PMC6076376 29908293

[B126] Zhang H. P. Liu X. L. Chen J. J. Cheng K. Bai S. J. Zheng P. (2020). Circulating microRNA 134 sheds light on the diagnosis of major depressive disorder. Transl. Psychiatry 10, 95. 10.1038/s41398-020-0773-2 32179735 PMC7075934

[B127] Zhao T. Piao L. H. Li D. P. Xu S. H. Wang S. Y. Yuan H. B. (2023). BDNF gene hydroxymethylation in hippocampus related to neuroinflammation-induced depression-like behaviors in mice. J. Affect Disord. 323, 723–730. 10.1016/j.jad.2022.12.035 36529411

[B128] Zhao Y. Sun L. Wang R. R. Hu J. F. Cui J. (2018). The effects of mitochondria-associated long noncoding RNAs in cancer mitochondria: new players in an old arena. Crit. Rev. Oncol. Hematol. 131, 76–82. 10.1016/j.critrevonc.2018.08.005 30293709

[B129] Zhu X. H. Qiao H. Du F. Xiong Q. Liu X. Zhang X. (2012). Quantitative imaging of energy expenditure in human brain. Neuroimage 60, 2107–2117. 10.1016/j.neuroimage.2012.02.013 22487547 PMC3325488

